# Seizures, Edema, Thrombosis, and Hemorrhages: An Update Review on the Medical Management of Gliomas

**DOI:** 10.3389/fonc.2021.617966

**Published:** 2021-03-22

**Authors:** Marco Zoccarato, Lucia Nardetto, Anna Maria Basile, Bruno Giometto, Vittorina Zagonel, Giuseppe Lombardi

**Affiliations:** ^1^ Neurology Unit, O.S.A., Azienda Ospedale-Università, Padua, Italy; ^2^ Neurology Unit, Trento Hospital, Azienda Provinciale per i Servizi Sanitari (APSS) di Trento, Trento, Italy; ^3^ Department of Oncology, Oncology 1, Veneto Institute of Oncology IOV-IRCSS, Padua, Italy

**Keywords:** BTRE, epilepsy, edema, hemorrhages, thrombosis, DOACs, glioma

## Abstract

Patients affected with gliomas develop a complex set of clinical manifestations that deeply impact on quality of life and overall survival. Brain tumor-related epilepsy is frequently the first manifestation of gliomas or may occur during the course of disease; the underlying mechanisms have not been fully explained and depend on both patient and tumor factors. Novel treatment options derive from the growing use of third-generation antiepileptic drugs. Vasogenic edema and elevated intracranial pressure cause a considerable burden of symptoms, especially in high-grade glioma, requiring an adequate use of corticosteroids. Patients with gliomas present with an elevated risk of tumor-associated venous thromboembolism whose prophylaxis and treatment are challenging, considering also the availability of new oral anticoagulant drugs. Moreover, intracerebral hemorrhages can complicate the course of the illness both due to tumor-specific characteristics, patient comorbidities, and side effects of antithrombotic and antitumoral therapies. This paper aims to review recent advances in these clinical issues, discussing the medical management of gliomas through an updated literature review.

## Introduction

Management of gliomas avails of multimodal and integrated curative options consisting of neurosurgical resection, chemotherapy (CT) and radiotherapy (RT). Despite recent therapeutic advances, including the introduction of novel anti-angiogenetic drugs like bevacizumab (1) and regorafenib (2), the illness course is associated with significant symptoms and complications that have a profound impact on quality of life and overall survival (3, 4).

Specifically, the control of seizures and symptoms related to peritumoral edema, and the treatment and prevention of hemorrhagic and thromboembolic events are core to the medical management of gliomas. These clinical issues are usually managed, along with curative treatments, by neuro-oncological multidisciplinary teams (5) requiring highly integrated, multi-professional expertise.

The relevance of supportive care in the management of gliomas in clinical practice is rapidly growing. This is due to the trend towards improvement in survival, the increasing incidence especially in the elderly (6) and the recent introduction of new therapeutic options which could be useful in supportive care, as third generation anti-epileptic drugs (AEDs) (7) for management of brain tumor-related epilepsy (BTRE), and direct-acting oral anticoagulants (DOACs) for the treatment of venous thromboembolism (VTE).

This review aims to summarize knowledge and suggest approaches to optimize medical management of gliomas drawn from recent literature.

## Tumor-Related Epilepsy

### Mechanisms and Risk Factors

BTRE contributes to morbidity and impacts on the quality of life of patients through the very occurrence of seizures, the side effects of medications and cognitive deterioration. Control of epilepsy therefore becomes paramount in the medical management of gliomas (8).

Mechanisms of BTRE have been partly explained and probably depend on intrinsic epileptogenicity (e.g., changes in neuro-transmitter balancing) and modification of the peri-tumoral environment (e.g., alteration of blood-brain barrier (BBB) and glial gap-junctional transmembrane communication proteins) especially in low grade tumors (9). High grade gliomas (HGG) determine epileptic seizures most probably through necrosis, hemosiderin deposition, hypoxia, and edema.

Young age (< 40 years old) (10) has been found to be independently associated with a higher risk of pre-operative seizures, whereas sex has apparently not influence (11, 12). More consistently, the incidence of BTRE depends on tumor-related variables, such as histotype, molecular profile, and location (**Table 1**).

**Table 1:** Risk factors for glioma manifestations and complications.

Tumor related epilepsy	Younger age Grading (LGG > HGG) Cortical localization (frontal and temporal lobe) IDH1 mutation	
Thromboembolic events (13, 14)	Patient-related factors Tumor-related Treatment-related	Older age (> 75) Obesity Prolonged immobility, leg paresis Prior thromboembolic events Multiple medical comorbidities A and AB blood type Grading (HGG > LGG) Intraluminal thrombosis (surgical specimens) Subtotal tumor resection Recurrent disease Tumor size (> 5 cm) IDH1 wild-type status Post-operative period Chemotherapy Anti-VEGF treatment (bevacizumab)
Hemorrhages	Patient-related Tumor-related Treatment-related	Hypertension Older age High degree of neo-angiogenesis Blood vessel invasion Tumor necrotic evolution Development of aneurysms Anticoagulation Radiotherapy and chemotherapy (depending on type and dose)

Frequency of seizures at onset and cumulative incidence during the illness are higher in low-grade gliomas (LGG) than in HGG. Seizures at the onset have been reported in up to 88% of LGG (15). Oligodendroglioma tends to confer a higher risk probably due to a higher cortical localization (16). In HGG frequency at the onset is 30 - 45% and cumulative incidence has been reported variably from 53% to 68% (11, 17).

Recently, association between glioma molecular markers and BTRE has been explored. Patients with isocitrate dehydrogenase 1 (IDH1) mutated gliomas have been found more likely to develop pre-operative seizures than wild-type IDH1 (18, 19); this could be due to the excitatory effect of the d-2-hydroxyglutarate product of mutant IDH1 on NMDA receptors (20). Other markers like expression of p53, ARTX loss, TERT mutation, 1p/19q co-deletion status or MGMT gene promoter methylation have not been found to correlate with pre-operative seizures (12, 21). As regards post-operative seizures, they could be associated with IDH1 mutation and MGMT methylation status (21).

Tumor location plays a significant role in conferring a higher risk of BTRE. The association with seizures is higher in cortical and juxtacortical tumors than in deep neoplasms (10, 22). The frontal lobe, especially the pre-motor area (23), temporo-mesial region and insula, are considered locations at higher seizure risk (24).

The volume of the glioma could also influence the risk of seizures. It was observed that HGGs presenting with seizures are usually smaller than those presenting with other symptoms, e.g., related to mass effect. Conversely, in the case of LGG, a larger tumor is more likely to present with seizures (22).

From a clinical point of view, seizures have a focal onset and may secondarily generalize. Not infrequently, gliomas can also manifest in the form of focal and generalized status epilepticus (25). Moreover, chronic continuous or sub-continuous focal seizures (*epilepsia partialis continua*) can develop during the course of illness (26); these seizures are often refractory to treatment.

### Management of BTRE

Seizures are responsive to conventional antitumor treatments. One of the aims of surgery is to improve seizures control. In LGG, seizures freedom one year after surgery is achieved in up to two thirds of patients with preoperative seizures (10, 16, 27). The extent of resection is a strong predictor of post-operative seizure outcome, as subtotal and total rather than partial surgical resections result in improved seizure control (27, 28) Additionally, patients aged > 45 years and patients with a history of seizures shorter than 1 year achieved better post-operative seizure control (29). RT also contributes to seizures control, and seizure-freedom at 12 months has been reported variably, from 32% to 75%, depending on timing, irradiation technique and tumor grading (30, 31). Prospective and retrospective series have shown that CT, either with temozolomide or PCV (procarbazine, lomustine, and vincristine) alone, have a beneficial effect on BTRE control (32, 33). Seizure improvement after the start of temozolomide could be a positive prognostic factor for progression free survival and overall survival (34).

Conversely, an antineoplastic role has been postulated for AEDs, which can directly contribute to improve overall survival (9). Several studies have shown that valproate, used during RT and the temozolomide course, improves overall survival (35–39). This could be due to a sensitizing effect to radiations as demonstrated *in vitro* (40). Notably, however, these results were not confirmed by a pooled analysis of more than 1800 patients involved in four randomized trials (41). An inhibitory effects on cell proliferation was shown for valproate and oxcarbazepine when tested *in vitro* on GBM cell lines (42), and for a number of third-generation AEDs like perampanel (43), brivaracetam, and lacosamide (44). Nevertheless, there is currently no clear evidence justifying the use of AEDs for reasons other than seizure control (45).

Prophylaxis with AEDs in patients with gliomas who have not developed seizures is generally not recommended (46, 47). In the perioperative and postoperative setting, prevention of early postoperative seizures is usually performed, although supporting evidence is limited (48, 49). Levetiracetam has been shown to be safer and more efficacious than phenytoin for this purpose (50). Optimal duration of postoperative prophylaxis is not well established; in clinical practice, according to AAN recommendations (46), it usually ranges from one to two weeks.

The choice of a specific AED in BTRE is not supported by large clinical trials. General recommendations adopted in the management of non-tumoral epilepsy should be applied. In case of a seizure, a monotherapy with the lowest effective dose should be started and the choice based on seizure type, age, sex, comorbidity, and side effects. There is a general consensus to avoid enzyme-inducing AEDs (e.g., carbamazepine, phenytoin, and phenobarbital) which increase the metabolism of chemotherapeutic agents and glucocorticoids. Notably, patients affected with gliomas are considered at higher risk than the epileptic population to develop side effects (51, 52). Seizures are usually treatment resistant and only half of patients respond to a single drug (53); during the course of illness, despite appropriate use of AEDs, up to half of patients continue to manifest seizures (54).

Currently, valproate is largely used to control seizures in gliomas due to its efficacy and possible positive influence on survival (10). Some concerns derive from side effects, especially the not infrequent hematologic toxicity during adjuvant temozolomide (36, 55). Levetiracetam is often chosen as the first monotherapy for its rapid titration and lack of interactions, with good efficacy and tolerability (56, 57). Neuropsychiatric effects, like anxiety, irritability or psychosis, are not uncommon, especially in frontal lobe tumors (58, 59). Recently lacosamide, a sodium channel blocker, has been increasingly reported as an add-on drug in BTRE with good efficacy and tolerability (60, 61). Other drugs approved for focal epilepsy have been tested in BTRE and can be an option especially for add-on therapy, like topiramate (62), pregabalin (63), and oxcarbazepine (64). Promising data in this setting are accumulating on the use of further third-generation AEDs like perampanel, an AMPA receptor antagonist, brivaracetam, a molecular analog of levetiracetam binding synaptic vesicle protein SV2A, and eslicarbazepine, an analog of carbamazepine with better interaction profile and moderate induction on CYP3A4 (**Table 2**).

**Table 2:** Third-generation antiepileptic drugs in BTRE (add-on therapy).

	Starting dose	Maintenance dose	Mechanism of action	Enzyme induction/inhibition°	Number of patients reported in BTRE (with references)
Lacosamide	50 mg bid	100 - 200 mg bid	Inactivation of voltage-gated sodium channels	None	70 (65), 14 (66), 71 (60), 16 (67), 25 (68), 93 (61), 105 (69)*, 39 (70)*
Perampanel	2 mg once daily	4 – 12 mg once daily	AMPA antagonist	Induces metabolism of progestin-containing contraceptives	36 (71), 26 (72), 8 (73), 12 (74)*, 11 (75)*
Brivaracetam	25 mg bid	25 – 100 mg bid	SV2A binding	Weak inhibition of CYP2C19 (↑ phenytoin levels) Inhibition of epoxide hydrolase (↑ carbamazepine epoxide)	33 (76)*
Eslicarbazepine	400 mg once daily	800 – 1600 mg once daily	Inactivation of voltage-gated sodium channels	Moderate induction of CYP3A4 (eg ↓ simvastatin and hormonal contraceptives). Weak inhibition of CYP2C19 (↑ phenytoin levels) ↓ warfarin levels (not clinically relevant)	8 (77)*

°Data from Lexicomp Online ^©^ 1978-2020 Lexicomp, Inc. All Rights Reserved. Accessed on October 14, 2020;

*Retrospective studies.

Discontinuation of therapy in patients who achieve freedom from seizures is a frequent and challenging clinical issue. Factors to consider before withdrawing AEDs are the severity of seizures, the anti-tumor treatments performed, the evaluation of side effects, and psychosocial impact (e.g., loss of drivers’ license in case of seizure relapse) (54). Two years after withdrawal, the recurrence rate in seizure-free patients with LGG and anaplastic glioma (more than 1 year from the last anti-tumor treatment) has been estimated at about one in four (78). Withdrawal should be avoided in the presence of tumor progression or recurrence (47). There is uncertainty as to the time of withdrawal; for glioma patients a minimum period of 1 year with seizure freedom and clinico-radiological stability could be appropriate (54).

While a recurrence of seizures in GBM patients with postoperative control of seizures should be considered as a marker of possible tumor relapse (47), this correlation is more controverted in LGG (54).

Electroencephalography (EEG) is useful for diagnosing a seizure related to a glioma when there is clinical uncertainty about the diagnosis, or for patients with nonconvulsive seizures (79). In glioma patients without seizures there are no data supporting the start of AEDs on the basis of EEG. Periodic EEG is not clearly useful and antiepileptic therapy modifications or withdrawal should not be based on EEG findings (47, 80) (**Table 3**).

**Table 3:** Brain-Tumor Related Epilepsy: key points and management.

The incidence of BTRE depends mainly on tumor-related variables, such as histotype, molecular profile, and location
Prophylaxis with AEDs is not recommended in patients with gliomas who have not developed seizures	
Prevention of early postoperative seizures should be stopped gradually one or two weeks after surgical intervention if the patient remains seizure free
Enzyme-inducing AEDs increase the metabolism of chemotherapeutic agents and glucocorticoids; accordingly they should avoided in the management of BTRE
For glioma patients, a minimum period of 1-year with seizure freedom and clinico-radiological stability could be appropriate before considering withdrawal of AEDs

## Vasogenic Edema

### Mechanisms

Gliomas are associated with vasogenic edema, which occurs when plasma-like fluid enters the extracellular space through an BBB driven by a hydrostatic pressure gradient. The BBB leak is probably due to a reduced numbers of normal astrocytes in tumor tissue and to an excessive secretion of angiogenetic factors such as vascular endothelial growth factor (VEGF) (81). Newly formed tumor vessels do not have mature tight junctions, and this leads to an increase in permeability (82). Tumor overexpression of the membrane water channel protein aquaporin-4 (AQP4), which regulates transcellular water movement and extracellular fluid resorption, has also been identified as a possible mechanism (83). The effects of brain edema include focal deficits and, through increasing intracranial pressure (ICP), headache, nausea, and vomiting.

### Management

Steroids are the mainstay of edema treatment, but their exact mechanism is not completely understood. Dexamethasone could decrease brain edema inducing restoration of normal BBB permeability mimicking the BBB-inducing properties of astrocytes (84). In addition, dexamethasone inhibits VEGF production and its effects on vessel walls (85). In clinical practice, steroids help to control peritumoral vasogenic edema and alleviate accompanying signs and symptoms. They also have antiemetic and analgesic effects and improve appetite and mood. In glioma patients the most common steroid used for brain edema is dexamethasone. It has little mineralocorticoid activity, probably a lower risk of infection and cognitive effects, a long half-life and high potency. There are no standardized guidelines for the timing, dose, duration and taper schedule of steroids (86). The conventional starting dose ranges between 8 and 16 mg/day (87). When administered twice daily, it is preferable to administer the second dose in the afternoon rather than in the evening to reduce the risk of insomnia (88). After surgical debulking, dexamethasone is usually tapered but more than half of patients require an increase in dose during RT to reduce ICP (89, 90). As a general rule, steroids should be used at the smallest effective dose and for the shortest period (91). Indeed, steroids have been associated with several adverse effects, with a cumulative effect (dose and duration of treatment): sleep disturbances, increased appetite and mood changes, osteoporosis and proximal myopathy, diabetes mellitus, accelerated atherosclerosis, gastrointestinal bleeding, T-cell mediated immunosuppression, and opportunistic infections (such as candidiasis) in addition to an important drug-to-drug interaction due to its metabolism through the CYP450 system (92).

Notably, corticosteroids can change the appearance of gliomas on MRI, with effects on the size of the contrast-enhancing tumor core and of peritumoral edema (88, 93), particularly for higher doses and longer therapy. Defining disease progression can be complicated during steroid treatment; the ongoing corticosteroid therapy is in fact one of the RANO (Response Assessment in Neuro-Oncology) criteria to evaluate treatment efficacy (94).

These data confirm that dexamethasone use should be limited to symptomatic patients and those with radiological findings of increased ICP. Moreover, it should be tapered to the lowest dose as soon as patient’s condition improve.

Alternative therapy or adjunctive therapy with a potential steroid-sparing effect have been considered. In patients with elevated ICP and consequent risk of herniation, mannitol and hypertonic saline, diuretics and fluid restriction, together with elevation of the bedhead and hyperventilation, help to rapidly reduce ICP in an emergency setting. On the other side, for long term control of edema and in refractory cases, antiangiogenic agents as bevacizumab (a monoclonal antibody against VEGF) and cediranib (a tyrosine kinase inhibitor of VEGF receptors) have been proposed (95–97). It has been demonstrated that bevacizumab and cediranib use is associated with a reduction in cerebral edema, as documented by MRI, with a significant reduction in steroid dose and neurologic improvement, although there is a rebound effect after therapy cessation. Conversely, severe cerebral edema following the use of immunotherapy agents such as nivolumab, an immune checkpoint inhibitor, has been reported (98) (**Table 4**).

**Table 4:** Edema: key points and management.

Corticosteroid therapy is not recommended in asymptomatic patients	
Dexamethasone with a starting dose of 8 – 16 mg/day (given as single dose or twice daily) should be administered depending on symptoms severity
Dexamethasone should be used at the smallest effective dose and for the shortest period
In case of elevated ICP due to edema and mass effect, mannitol and hypertonic saline, diuretics, and fluid restriction can be administered

## Venous Thromboembolism

### Risk Factors and Biomarkers

Patients with HGG present with the highest risk of tumor-associated VTE out of all cancer patients, with observed rates as high as 7.5-39% depending on the use of thromboprophylaxis and the detection method (13, 99–102). The observed incidence of VTE was reported to be 8% in a large retrospective study (103), and 21–26% at 1 year and 32% at 2 years in prospective studies (100, 104).

Outcome data for malignant glioma patients are similar to those for cancer patients; a large neurosurgical cohort showed that patients with VTE had a 30% higher risk of death within 2 years (HR 1.3; CI 1.2–1.4) compared to those without VTE (103).

VTE risk factors in glioma patients can be grouped into patient-, tumor-, and treatment-related risk factors (**Table 1**). Among patient-related risk factors, leg motor impairment has been consistently reported, with a relative risk for VTE of between 2.6 and 3.6 (104, 105). Other independent patient-related factors include age greater than 75 years (105), elevated BMI, and A or AB blood type (106). Tumor-associated factors include tumor grade (HGG vs LGG) and tumor size greater than 5 cm (103, 107), subtotal surgical resection as compared with total resection (100), intraluminal thrombosis in the surgical specimen (108, 109), recurrent disease, and IDH-1 wildtype glioma (110).

CT and anti-VGEF agents increase VTE risk in glioma (111–113). RT predisposes patients to VTE in other cancers, while there are no similar data available for brain tumors. Likewise, corticosteroids are associated with increased rates of VTE in other tumors; however, the role of corticosteroids as an independent risk factor for VTE in malignant glioma patients remains undefined. Bevacizumab is the first approved antiangiogenic therapy for recurrent GBM. A well-documented side effect of bevacizumab in extra-central nervous system (CNS) malignancies is intratumoral bleeding. Bevacizumab has been linked to an increased risk of arterial and venous thromboembolic events in other cancers too. Few data are available regarding the concomitant use of bevacizumab and curative anticoagulants in GBM patients with a VTE. Nevertheless, the risk-to-benefit ratio seems to favor this combination despite an increased risk of intracerebral hemorrhage (ICH) (from 3 to 11%) (112).

Biomarkers suitable to assess the VTE risk in HGG patients were recently described: platelet count, D-dimers, sP-selectin levels, factor VIII activity, prothrombin fragment 1 + 2, and leucocyte count. The authors also identified high-risk and low-risk patients with two risk assessment models (114).

### Mechanisms

A central role in the pathogenesis of VTE in gliomas has been suggested for tissue factor (TF) (115), the major activator of the coagulation system, by initiating the extrinsic pathway of the coagulation cascade. This protein is expressed on perivascular cells such as fibroblasts and vascular smooth muscle cells, but it can be expressed also by tumor cells, activated leucocytes, and endothelial cells. In addition to its crucial role in coagulation, TF has a shorter cytoplasmic domain, which mediates several downstream signaling effects, including activation and upregulation of VEGF (116), suggesting a role in cancer growth and angiogenesis. Constitutive over-expression of TF has been shown in glioma (117) and correlates with glioma grade (118) Also TF bearing microparticles, detectable in the plasma samples from cancer patients including those with GBM, may play a role in cancer-associated VTE (115, 119).

Very recently, a role in cancer-related VTE has been suggested for prodoplanin (115) which is a ligand of the C-type lectin-like receptor 2 on platelets (CLEC-2) and induces platelet aggregation. An overexpression of podoplanin has been shown exclusively in IDH1-wild-type brain tumors (14). On the contrary, the risk of VTE has been reported to be extremely low in patients with IDH1 mutated gliomas (110).

### Prophylaxis of Venous Thromboembolism

An assessment of VTE risk based on a validated assessment tool is crucial in cancer patients (120, 121). In the general cancer population, pharmacological thromboprophylaxis with low-molecular-weight heparin (LMWH) is recommended in hospitalized patients and in the perioperative setting (120). This recommendation also applies to HGG patients, notwithstanding that the risk of ICH in these patients limits the use of pharmacological approaches to VTE prevention. In neurosurgical patients, a clinical trial of medical prophylaxis using unfractionated heparin (UFH) or LMWH combined with a compression stocking showed superiority in VTE prevention to a mechanical device alone (122). A meta-analysis (123) in the controversial setting of neurosurgery, showed a 45% relative risk reduction of VTE in favor of UFH/LMWH. Adverse bleeding, including major and intracerebral bleeds, was increased in the heparin arm but the absolute risk was low and not considered by expert consensus to be clinically relevant. In these studies, UFH or LMWH was started post-operatively, usually 24 hours after surgery. It has been suggested that delaying to 48 hours is associated with a 25% increase in the risk of thrombosis, whereas preoperative administration of LMWH leads to an excess of intracerebral bleeding (124). The timing of pharmacological prophylaxis may be therefore be crucial.

For patients undergoing major surgery for cancer, guidelines recommend continuing pharmacologic thromboprophylaxis for at least 7 to 10 days (120). In brain tumor patients VTE prophylaxis with LMWH should be started within 24 h after surgery (4).

Although the risk of VTE remains high throughout the course of the disease, to date no study has demonstrated an advantage from prolonging prophylaxis beyond the perioperative period. A placebo-controlled trial, the PRODIGE study (125), aimed to evaluate the efficacy and safety of primary thromboprophylaxis with LMWH (dalteparin) for up to 12 months in patients with malignant glioma. It showed a non-significant trend toward a reduced risk of VTE in the LMWH group after 6 months of therapy. A trend toward increased risk of major bleeding after 12 months was instead seen, and all major bleeds were ICH.

### Treatment of Venous Thromboembolism

There is no standardized approach to the management of glioma patients suffering from a VTE because most existing guidelines address the general cancer population. Patients with brain tumors have unique management issues, including fear of ICH, medication compliance, and common drug-drug interactions. Despite fears of ICH, it appears to be safe to offer full therapeutic anticoagulation to patients with brain tumors presenting with a VTE (13, 101). In HGG, the risk of spontaneous hemorrhage (ICH) has been reported to be between 2% and 8%, with higher rates in GBM and anaplastic oligodendroglioma (126). Al Megren and collegues (127) reported a seven-fold increase in the risk of ICH in glioma patients who received full dose anticoagulation for acute VTE treatment as compared with glioma patients without VTE. In a meta-analysis of nine retrospective cohort studies (128) a 3-fold higher risk of ICH in glioma patients receiving therapeutic anticoagulation was reported. Postoperative blood products in asymptomatic patients do not constitute an absolute contraindication to anticoagulant use for proven symptomatic VTE. On the contrary, curative doses of anticoagulation should be avoided in patients with recent symptomatic intratumoral bleeding, thrombocytopenia under 50000 platelets/mm^3^, and other usual contraindications.

Most guidelines recommend LMWH over vitamin-K antagonist for treatment of acute VTE in cancer patients (129–131). In the most updated American Society of Clinical Oncology (ASCO) practice guidelines (132), DOACs were added as options for VTE prophylaxis and treatment in cancer patients. Anticoagulation should be initiated as soon as possible after diagnosis of VTE. This recommendation was mainly based on clinical randomized controlled trials testing initial therapy with LMWH followed by either warfarin or LMWH for a total of 6 months of therapy (133), with better efficacy being reported for LMWH over VKA for treatment of VTE in cancer patients. LMWH appears to be well tolerated in the curative setting of patients with HGG, showing several clinical advantages compared to warfarin, including no need for laboratory monitoring, and minimal drug and food interactions. However, despite these advantages, daily subcutaneous injections and cost represent a barrier to widespread use. Many HGG patients, particularly those compliant with warfarin dosing and monitoring who are not on conflicting medications, and those with significant renal impairment, may be safely managed with warfarin, acknowledging some increased risk of recurrent thrombosis. Very recently, a few studies investigated a possible role for new oral anticoagulants in this setting. The main concerns include potential drug interaction with CT and antiepileptic agents. The Hokusai VTE-cancer study (134) showed that oral edoxaban was non-inferior to subcutaneous dalteparin with respect to the composite outcome of recurrent VTE and major bleeding during 12 months after randomization in the general cancer population. The rate of recurrent VTE was lower but the rate of major bleeding, particularly gastrointestinal bleeding, was higher with edoxaban than with dalteparin. Efficacy and safety did not differ between treatment arms also in a subgroup analysis of patients with brain tumors. In the SELECT-D study (135), the anti-Xa-inhibitor rivaroxaban was compared with dalteparin. Rivaroxaban was associated with relatively low VTE recurrence, no increased risk of major bleeding events, but higher risk of clinically relevant non-major bleeding as compared with dalteparin. However, in this study the number of included patients with brain tumors was very small.

Current data suggest similar efficacy and safety for edoxaban as compared with dalteparin for VTE treatment in patients with brain tumors. However, evidence is so far very limited, and further studies are warranted. According to the current evidence, the most updated ASCO practice guidelines (132) recommend anticoagulation for patients with primary or metastatic brain malignancies and an established VTE, although uncertainties remain about the choice of agents and selection of patients most likely to benefit. All recommendations regarding the insertion of a vena cava filter were made with informal consensus and low-quality evidence. It may be offered to patients with absolute contraindications to anticoagulant therapy, but it is associated with a high failure rate of up to 62% of patients with brain tumors.

The duration of secondary prophylaxis in patients with gliomas after a venous thromboembolism event should be planned individually, weighing the risk of ICH against the risk of VTE recurrence in patients whose tumor cannot be considered stable. However, it is lifelong in most patients (4) (**Table 5**).

**Table 5:** Venous thromboembolism: key points and management.

In patients with gliomas, pharmacological thromboprophylaxis with low-molecular-weight heparin (LMWH) is recommended in hospitalized patients and in the perioperative setting.
Thromboprophylaxis with LMWH should be started within 24 h after surgery and continued for at least 7 to 10 days.
Anticoagulation is recommended in case of established VTE even if an increased risk of ICH is reported.
There is no clear evidence on the superiority of LMWH or DOACs in the treatment of VTE.

## Intracerebral Hemorrhages

### Mechanisms

Patients with gliomas are at risk for hemorrhagic complications, in particular for ICH. Gliomas present distinctive mechanisms of ICH that differ from those of the general population (136–138). Although patients can present with subdural or subarachnoid hemorrhages (caused by neoplastic aneurysms or meningeal spreading), most cases of ICH in glioma patients are intratumoral bleedings (139, 140). It is deemed that approximately 2 to 5% of gliomas can develop an intralesional hemorrhage, especially in GBM, followed by oligodendrogliomas and astrocytomas (136, 141). The tendency of a glioma to develop intralesional bleeding is considered to be related to degree of angiogenesis, development of aneurysms, blood vessel invasion, and consequent risk of rupture, and to tumor necrotic evolution. In particular, angiogenesis is a process driven mainly by vascular endothelial growth factor (VEGF): the newly formed tumor vessels differ from normal cerebral vasculature because they lack tight junctions and, consequently, a mature BBB; this renders them fragile and susceptible to rupture and bleeding (136).

### Diagnosis and Risk Factors

**Figure 1:**
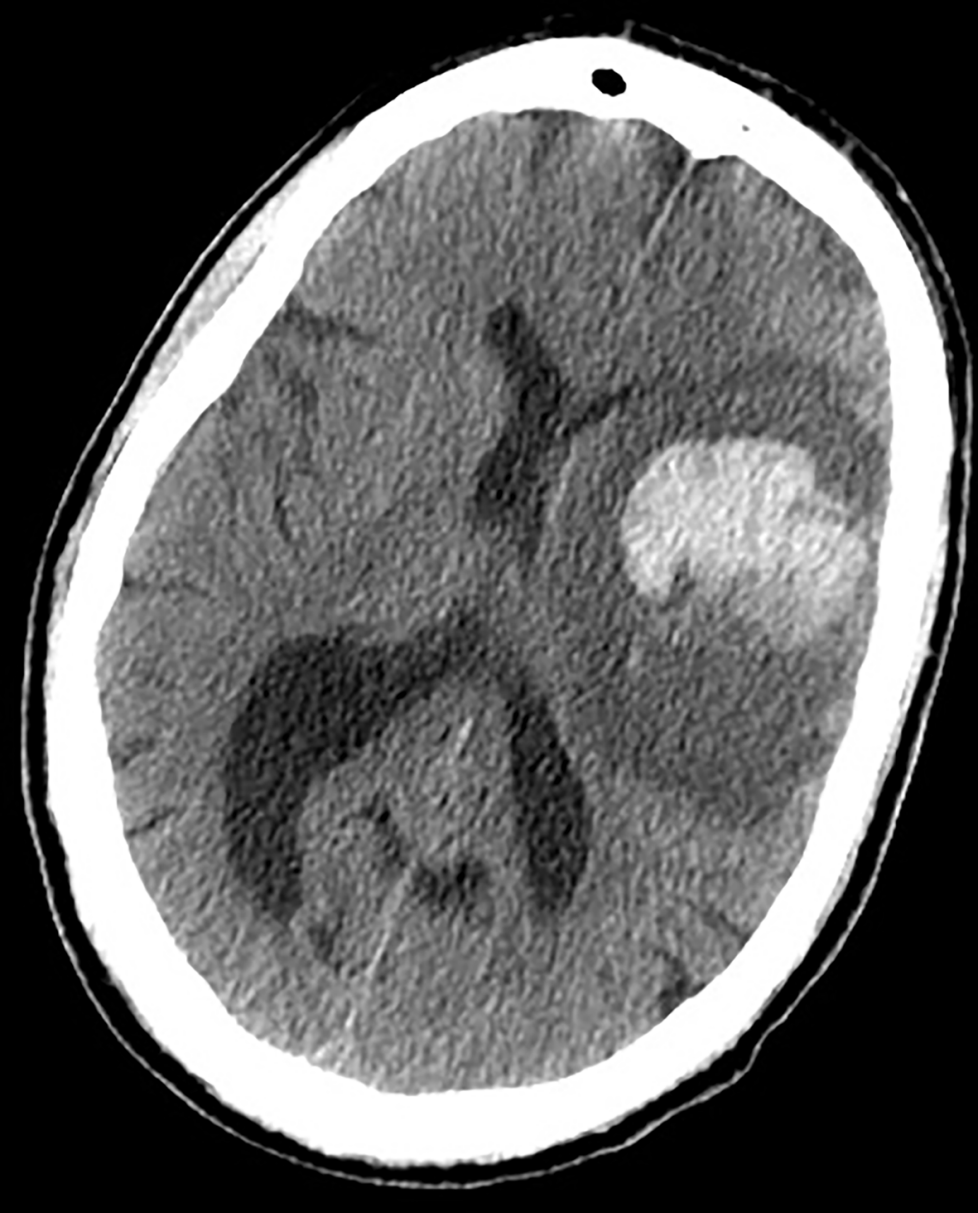
Earlier brain CT scan of a 71-year-old man presenting with acute onset of right hemiparesis: note the disproportionate hypodense edematous area surrounding the hematoma, not in accordance with the very acute phase of hemorrhage. An underlying unknown HGG was diagnosed.

**Figure 2:**
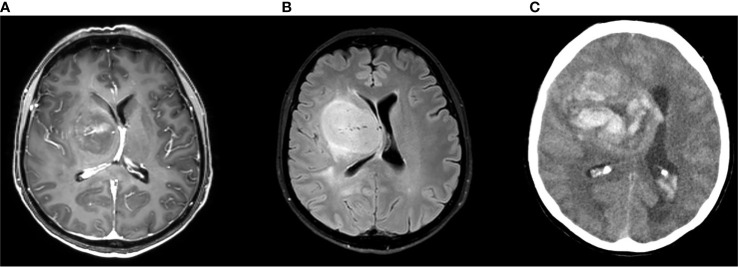
Brain MRI scan (**A**: gadolinium enhanced image **B**: FLAIR image) of a 53-year-old woman complaining of subacute onset of mild left hemiparesis showing highly vascularized HGG; 8 days later the patient developed headache and brain CT scan **(C)** showed a wide intralesional bleeding.

ICH can be the first manifestation of an unknown brain tumor (**Figure 1**) or a complication during neoplasm treatment or follow-up (**Figure 2**). In the former case, ICH causes acute onset of focal neurological deficits which are indistinguishable from those caused by spontaneous ICH. If not promptly differentiated, intralesional bleeding can delay neoplasm diagnosis and treatment (142). Primary brain tumors, mainly gliomas, account for 2 to 6% of all ICH cases (143–145). Differentiating intratumoral bleeding from more common causes of ICH may be challenging and the differential diagnosis is based on anamnestic and radiological evaluations. In the case of ICH, an underlying tumor should be suspected when a history of preceding headache or focal neurological deficit or personality changes for days/weeks prior to the onset of ICH is reported. Neuroradiological characteristics of glioma-associated ICH that help in the differential diagnosis are a) ICH affecting structures that are rarely involved in hypertensive ICH (such as the corpus callosum, which in turn is frequently affected by GBM); b) an area of “ring-like” hemorrhage with a low-density center in a non-contrast computed tomography scan; c) multiple stages of hematoma in the same lesion. Probably the most characteristic aspect of glioma-related ICH is perihematomal edema. Indeed, edema and mass effect are usually a late phenomenon in spontaneous ICH and develop gradually, but are usually present in the very first computed tomography scan in the case of intratumoral bleeding. In other words, tumor hemorrhage does not usually correspond to hematoma age, rather it shows a premature and disproportionate degree of surrounding edema and mass effect (**Figure 1**). In most cases of ICH, clinical and radiological follow up is required to confirm diagnosis (146).

When ICH occurs during glioma treatment, it worsens patient conditions, pre-existing deficits and prognosis. ICH can be a complication of CT, RT or other treatments. For instance, VTE treatment usually requires anticoagulation but the management of anticoagulation in these patients is challenging because of the high risk of both recurrent thrombosis and hemorrhagic complications, in particular ICH (see above).

Since glioma treatment, as brain RT and CT, may alter the structure and integrity of the intracranial vessels, it has been associated with an increased risk of both ischemic and hemorrhagic cerebral events. Specifically, RT plays an important role in glioma management but its involvement in accelerating atherosclerosis is well recognized, as is its influence in the development of aneurysms, teleangectasias, and cavernomas in pediatric primary brain tumors treated with RT (147–149). These vascular malformations may in turn rupture causing ICH. ICH other than intralesional bleeding (i.e., caused by the rupture of a vascular malformation) seems to be associated with RT dose, tumor location, CT type and dose, and age, with a median interval between RT and ICH development of 8.1 years (149, 150). Moreover, apart from their role in promoting the development of vascular malformations, animal model, autoptic, and radiological studies have demonstrated radiation-induced microvascular damage, endothelial degeneration, and the formation of cerebral microbleeds (151, 152): these processes progressively increase with each passing year from time of RT and are responsible for increased, late, cerebral hemorrhagic risk, but also for cognitive decline.

Considering the effect of CT on hemorrhagic risk in glioma patients, a possible negative influence has been attributed to bevacizumab, a recombinant humanized monoclonal antibody directed against VEGF and used for the treatment of recurrent and progressive glioblastoma multiforme (1, 153, 154) Despite its efficacy in increasing progression-free survival and in improving symptoms, early clinical studies have shown a possible increased risk of hemorrhage within and adjacent to the tumor, and a history of ICH is a relative contraindication to bevacizumab therapy (155, 156). ICH risk can vary according to dose, concomitant RT, patient characteristics, and therapies. (**Table 1**) However, in randomized phase 3 study evaluating the safety and efficacy of bevacizumab plus lomustine in 288 recurrent glioblastoma patients, only one case (0.3%) reported bevacizumab-related ICH, demonstrating the low incidence of this adverse event during bevacizumab treatment (157).

Conversely, regorafenib demonstrated to improve both progression-free and overall survival in recurrent glioblastoma patients (2); regorafenib is an oral multityrosine kinase inhibitor interfering with angiogenesis, oncogenesis, and tumor microenvironment. Use of regorafenib is not related to the increased incidence of ICH or VTE in glioma patients; the most frequent adverse events are hand-foot syndrome and alteration of liver function.

Currently there is not specific treatment for glioma-related hemorrhages. If surgical management is not required, edema treatment and blood pression control are the main therapy goals (158). In patient bleeding while on anticoagulants, appropriate reversal of the anticoagulant effect should be initiated according to current guidelines (159) (**Table 6**).

**Table 6:** Intracerebral hemorrhages: key points and management.

ICH risk in glioma patients is related to specific tumor-related mechanisms, to patient vascular risk factors and comorbidities, and to therapy (CT, RT, and antithrombotic therapy).
The most important cause of suspicion in differentiating spontaneous vs intratumoral cerebral bleeding is perihematomal edema.
Anticoagulation is associated with a significant increase in the rate of ICH in glioma patients
If surgical management is not required, edema treatment and blood pression control are the main goals.
In patient bleeding while on anticoagulants, appropriate reversal of the anticoagulant effect should be initiated according to current guidelines

## Discussion and Conclusion

This paper reviewed some important symptoms and complications of gliomas, focusing on epilepsy, edema, and thromboembolic and hemorrhagic events. Further relevant clinical issues also commonly arise in supportive care, as the management of cognitive deficits, fatigue, mood disorders, and hematological complications of CT. These aspects need to be carefully prevented and treated since failure to control them can negatively influence patient survival and quality of life. Notably, adequate medical management can prevent avoidable hospital admissions, determining higher healthcare costs (160). Therefore, neuro-oncological patients need regular follow up also after the end of the curative therapies in order to address important social and medical needs (161). This result can be achieved by an integrated approach in multi-professional teams.

The quality of available evidence on these topics is low and several points are yet to be clarified. For instance, there is still an open discussion on the role of prophylactic AED use, which most guidelines do not recommend. The majority of studies supporting this approach were performed with older AEDs. Recently released AEDs could reduce potential harm deriving from adverse effects and drug-drug interaction and make the risk/benefit ratio more favorable. Conversely, there is no evidence that they are more effective than older AEDs in preventing seizures in glioma patients. At the same time, routine use of perioperative prophylaxis, although frequent, is based on limited evidence (79). In the coming years, advances in the management of BTRE could be derived from large trials, including comparison trials, to assess the benefit of AEDs in BTRE. In addition, the antitumoral activity of AEDs, the impact of their use on overall survival, and the correlation between drug responsiveness and glioma molecular markers should be tested through large observational studies (162).

Thromboembolic events in cancer patients, including gliomas, are a topic of growing interest and increasingly the focus of studies and guidelines. Since DOACs have shown a significant reduction in the risk of intracerebral bleeding compared with vitamin k antagonists in patients with both atrial fibrillation and VTE, the idea that the same advantage could be present in subjects with brain tumors is reasonable. However, the number of patients with brain tumors included in recent trials to test the use of DOACs in cancer patients with established VTE was very small, and the risk of intralesional bleeding in patients with gliomas undergoing anticoagulant therapy with said agents has not yet been established. More trials are needed to define the anticoagulant agent with the best efficacy and safety profile in this subgroup of cancer patients.

The risk of ICH in glioma patients is not only related to anticoagulation and intralesional bleeding but is encountered throughout the whole illness trajectory. Future studies could establish if there are radiological and molecular markers that could better estimate ICH risk and stratify the glioma population in order to adjust therapies and render these very severe events preventable. Moreover, the influence of antiangiogenetic drugs on this risk should be better assessed.

## Author Contributions

MZ, LN, and AB conceived and drafted the review. BG, VZ, and GL revised the manuscript for important intellectual content. All authors contributed to the article and approved the submitted version.

## Conflict of Interest

The authors declare that the research was conducted in the absence of any commercial or financial relationships that could be construed as a potential conflict of interest.
